# A flexible simulation platform to quantify and manage emergency department crowding

**DOI:** 10.1186/1472-6947-14-50

**Published:** 2014-06-09

**Authors:** Joshua E Hurwitz, Jo Ann Lee, Kenneth K Lopiano, Scott A McKinley, James Keesling, Joseph A Tyndall

**Affiliations:** 1 Department of Mathematics, University of Florida, Gainesville FL, USA; 2 Statistical and Applied Mathematical Sciences Institute, Research Triangle Park NC, USA; 3 Department of Emergency Medicine, University of Florida, Gainesville FL, USA

**Keywords:** Simulation, Emergency department, Throughput, Crowding, Quantify, Hospital, Site-specific, Boarding times, Fast track

## Abstract

**Background:**

Hospital-based Emergency Departments are struggling to provide timely care to a steadily increasing number of unscheduled ED visits. Dwindling compensation and rising ED closures dictate that meeting this challenge demands greater operational efficiency.

**Methods:**

Using techniques from operations research theory, as well as a novel event-driven algorithm for processing priority queues, we developed a flexible simulation platform for hospital-based EDs. We tuned the parameters of the system to mimic U.S. nationally average and average academic hospital-based ED performance metrics and are able to assess a variety of patient flow outcomes including patient door-to-event times, propensity to leave without being seen, ED occupancy level, and dynamic staffing and resource use.

**Results:**

The causes of ED crowding are variable and require site-specific solutions. For example, in a nationally average ED environment, provider availability is a surprising, but persistent bottleneck in patient flow. As a result, resources expended in reducing boarding times may not have the expected impact on patient throughput. On the other hand, reallocating resources into alternate care pathways can dramatically expedite care for lower acuity patients without delaying care for higher acuity patients. In an average academic ED environment, bed availability is the primary bottleneck in patient flow. Consequently, adjustments to provider scheduling have a limited effect on the timeliness of care delivery, while shorter boarding times significantly reduce crowding. An online version of the simulation platform is available at http://spark.rstudio.com/klopiano/EDsimulation/.

**Conclusion:**

In building this robust simulation framework, we have created a novel decision-support tool that ED and hospital managers can use to quantify the impact of proposed changes to patient flow prior to implementation.

## Background

### Introduction

Hospital-based Emergency Departments are struggling to provide timely care to a steadily increasing number of unscheduled ED visits [1]. Dwindling compensation [2] and rising ED closures [3] dictate that meeting this challenge demands greater operational efficiency. However, when compared to other areas within the healthcare system, EDs present a unique environment of competing priorities, limited resources, and a wide variety of patients demanding care.

Understanding the complexity of such environments requires more than experience and intuition alone. There is a growing consensus that effective management of care delivery in hospital-based EDs requires the support of mathematical and computational modeling. Indeed, the primary recommendation of the Institute of Medicine’s 2006 report, *Hospital-Based Emergency Care: At the Breaking Point* was the development of engineering and operations research tools for the purposes of improving ED efficiency and increasing patient flow.

In recent years, there have been many efforts in this direction [4]. Mathematical and computational models have been used to forecast ED crowding on a scale of hours [5], quantify factors contributing to patients leaving without being seen (LWBS) [6-9], assess patient streaming mechanisms [10-15], optimize staff and resource allocation [16-21], conduct financial analyses [22-24], and study the impact of reducing boarding times [18,23]. However, because it is risky to implement management overhauls, a gap remains between ED models and current management practice [25].

### Importance

ED management face a variety of options when deciding how to improve efficiency, and seemingly straight-forward operational innovations can be rendered ineffective by counterintuitive patient flow dynamics [18,23,26]. The utility of patient flow simulations lies not in simplifying this complexity, but in capturing it. A detailed model of ED throughput can accurately quantify predictions for management interventions that are formulated by experience and intuition.

### Goals of this investigation

The first goal of this investigation was to develop a widely-configurable discrete-event simulation framework that allows for quantification of long-term patient flow outcomes. The second goal was to validate the ability of the model to accurately simulate two distinct ED environments – one resembling a nationally average ED and one resembling an average academic ED, both in the United States. The third goal was to simulate and analyze the addition of beds and staff, the implementation of alternate care pathways, and reductions in boarding times in each of these environments.

## Methods

### Patient flow model

**Figure 1:**
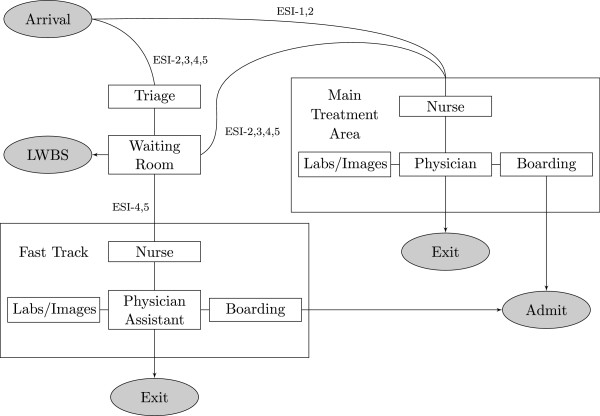
**Patient flow map.** Patient paths are directed based on acuity and resource needs. If an ED does not utilize a Fast Track, all patients are assigned a bed in the Main Treatment Area.

To construct a map of patient flow through an ED (Figure 1), we conducted in depth interviews with providers regarding work processes and operational characteristics of an example ED. We assume the following patient flow structure: Upon arrival to the ED, patients are streamed according to their Emergency Severity Index (ESI) score [27]. The most acute patients are immediately classified as ESI-1 and are taken directly to a trauma/resuscitation bed in the Main Treatment Area (MTA) of the ED where their treatment preempts that of lower acuity patients currently being treated. A fraction of ESI-2 patients also bypass triage and go directly to an MTA bed. All other patients receive an ESI score between 2 and 5 in triage and move to the waiting room until a bed becomes available. Patients in the waiting room are selected for bed assignment based on acuity and time of arrival. In some of our experimental scenarios, there is a separate Fast Track (FT) area available for use by ESI-4 and ESI-5 patients. Patients who stay too long in the waiting room (i.e. who do not receive a bed before their tolerance for waiting) leave without being seen.

Patients who do not leave are assigned to an MTA or FT bed, and are briefly assessed by a nurse. A history is taken and a physical exam is then performed by a physician; the physician might subsequently order labs or radiological testing, perform procedures, or disposition the patient. Patients who have labs or images ordered occupy a bed and receive intermittent nursing attention until the results are ready and a physician returns to review them; the physician can then order more tests, perform procedures, or disposition the patient. In the FT, physician assistants (PAs) perform the duties of physicians. In both treatment areas, patients who are dispositioned to discharge exit the ED after a short delay to receive discharge instructions; patients dispositioned to admit remain in their assigned bed and receive care until a hospital bed is available – a process known as boarding.

#### Simulation details

The simulation platform code is written in R and utilizes stochastic, event-driven programming to model patient flow through a user-configured ED environment.

In our model, acuity – as measured by ESI – is the primary determinant of patient complexity, streaming, and prioritization. As such, parameters governing patient arrival rates, nurse-to-patient ratios, tolerance before LWBS, treatment steps, admit rates, and boarding delays are modulated to reflect this acuity-dependence.

ED dynamics are intrinsically variable and so any model ED must embrace randomness as a core feature. Unfortunately, most of the data available for event durations only include measures of central tendency. To account for natural variation in event durations, we used independent Gamma-distributed random variables. This was motivated by the fact that Gamma random variables are completely characterized by their mean and variance, and represent the waiting time between multiple Poisson-distributed events.

**Figure 2:**
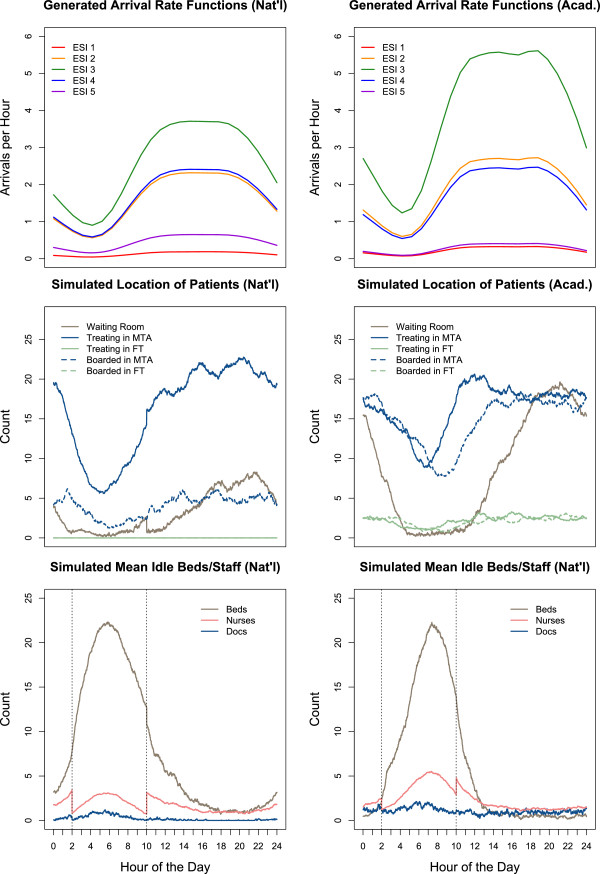
**A day in the life of an ED.** Generated arrival functions (top), and 30-day simulated location of patients (middle) and idle resources (bottom) for nationally average and average academic ED settings. The nationally average setting is limited by providers, while beds are the primary bottleneck in the average academic setting.

The most notable exception to the Gamma distribution framework is in patient arrival times, which are random and fluctuate depending on the time of day. We model patient arrivals by a non-homogeneous (time-dependent) Poisson process [28] – a stochastic process uniquely defined by its time-dependent arrival intensity. To create this intensity function, we used a step function generated from hourly ED arrival rate data [29] (Figure 2). These arrival rates were adjusted to account for the contribution from each acuity level – that is, a separate function was generated for each acuity’s arrivals. We note that arrival intensity functions for each acuity can be adjusted to fit data from any emergency department.

### Data resources

Publicly available data [29-31] provided many key parameters governing patient arrivals and complexities, boarding delays, and recommended staffing levels for the nationally average setting. With permission from the Academy of Academic Administrators in Emergency Medicine (AAAEM), we used data from a 2012 benchmark survey [32] to estimate these parameters for the average academic setting. ED providers estimated finer parameters such as lab and imaging turnaround times and patient-physician interaction lengths for both settings. Table 1 outlines the key input parameters for each ED environment and the output values used to validate the accuracy of each model. We stress that the data compared to the simulation outputs in the bottom of Table 1*was not used to construct either model* – rather, this data was used only to validate the accuracy of each model.In comparing nationally average statistics to data collected from academic hospitals, it is immediately clear that academic environments on average experience higher patient arrival rates. Moreover, the mix of patients tends to be more acute in academic settings (see top two panels of Figure 2). Because of this – and the fact that they are typically associated with large hospitals – academic EDs have higher admit rates and longer boarding times relative to the national average. To accommodate this, academic EDs tend to have more beds and higher staffing levels.

**Table 1:** Input parameters and output validation for the nationally average and average academic environments

**Input parameters**	**National average**		**Academic average**	
MTA Beds	32^1^		41^5^	
FT Beds	0^1^		6^2^	
Physicians	3^2^		4^2^	
PAs	0^2^		1^2^	
Nurses	8^2^		13^2^	
Nurse to Patient Ratio^*†*^	1:4^3^		1:4^3^	
Mean Arrivals per Day	155^1^		195^5^	
Mean LWBS Threshold^*†*^	3.5 hrs^2^		3.5 hrs^2^	
Mean Nursing Assessment^*†*^	5 min^2^		5 min^2^	
Mean Physician Assessment^*†*^	10 min^2^		10 min^2^	
Mean Lab TAT^*†*^	30 min^2^		45 min^2^	
Mean Imaging TAT^*†*^	75 min^2^		90 min^2^	
Admit Rate^*†*^	12.8%^1^		25.8%^5^	
Mean Boarding Delay^*†*^	1.63 hrs^4^		4.43 hrs^5^	
**Outputs**	**Simulated**	**Actual**^**1**^	**Simulated**	**Actual**^**5**^
LWBS Rate (%)	3.06 (1.03)	3.00	4.54 (0.88)	4.50
Door-to-Event Time (hrs)	—	—	—	—
*Doctor*	0.98 (0.10)	0.97	1.25 (0.07)	1.31
*Disposition*	3.06 (0.15)	3.08	3.41 (0.08)	3.41
*Exit*	3.71 (0.15)	3.73	5.63 (0.10)	5.67
Patients/Doctor/Hour	2.41 (0.02)	1.8-2.8^3^	2.21 (0.01)	2.51
Patients/Bed/Year	1764.5 (16.8)	no data	1513.8 (9.67)	1360.3

Input parameters for the nationally average and average academic environments (above). Simulated outputs reported as mean (sd) of 30 one-month simulations closely approximate actual data (below). Note: between 2:00am and 10:00am, the number of physicians is reduced by 1 and the number of nurses is reduced by 3. ^*†*^These parameters are modulated to be acuity-dependent according to an ED provider heuristic; the figures reported are the mean over all patients. *TAT*: turnaround time. Sources: ^1^CDC [29], ^2^ED Provider Estimate, ^3^ACEP [31,34], ^4^CMS [30], ^5^AAAEM Survey [32].

Since all data used in this experiment was publicly available, approval by an ethics committee was not required.

### Limitations

Our model makes no assumptions regarding factors contributing to triage or registration delays. Instead, simulated patients are assigned a length of time drawn from a Gamma distribution to complete triage and registration. Because our model assumes short door-to-triage times, we assume any error is negligible; it is also computationally efficient to assume this distribution is state- and time-invariant.

While a patient’s decision to leave without being seen is influenced by many factors [33], our model also assumes that each patient’s decision to LWBS depends *only* on waiting time. To that end, each simulated patient arrives with a tolerance for waiting that is drawn from an acuity-dependent Gamma distribution. If a patient does not receive a bed before their tolerance for waiting, they exit the ED and are marked as LWBS.

Topology and layout are important factors that affect ED throughput. Our model accounts for this by incorporating the time it takes for a provider to move from room to room. This is modeled using time- and provider-dependent exponential random variables.

A more significant limitation is that our model assumes that physicians are not assigned to specific patients. Rather, when patients demand a physician, any available physician can provide care. In the academic environment, physician-providers typically work in teams of faculty paired with residents. For the purposes of this simulation, we assume that a single physician in the academic setting adequately represents a physician-resident team. We note that the simulated patients per doctor per hour statistic (Table 1) is a consistent value in both settings [32,34]. The development of an efficient algorithm to assign physicians to specific patients will improve ED simulations and deserves further study.

Nursing attention in an ED is a much more varied and continuous process than patient interactions with a physician. Rather than model patients’ demand for nursing care as multiple discrete intervals, simulated patients occupy a fraction of a nurse (corresponding to nurse-to-patient ratios) at all times while in an ED bed. Our model for management assumes that a patient cannot be placed in a bed without sufficient nursing staffing. Therefore, in our model, a nursing shortage will manifest itself as a lack of usable beds.

Boarding times in a real hospital setting are dependent on many factors outside of the ED, such as hospital capacity, transport efficiency, and discharge schedules. Our model does not simulate these directly. Rather, when a simulated patient is dispositioned to admit, a Gamma distribution is generated and a boarding time drawn from this distribution is assigned to the patient. The model allows for acuity- and time-dependent distributions, but due to a lack of concrete data, the simulations we report here used a boarding time distribution that was acuity- and time-invariant.

## Results

As is to be expected with a simulation of this magnitude, there are a very large number of parameters. In particular, we focused on two parameter regimes: one dictated by a lack of providers, the other by a lack of beds. Interestingly, equipping the system with nationally average statistics led to provider-limited dynamics, while using average academic hospital statistics led to bed-limiteddynamics.

The results flow from three phases of analysis: *validation*, *explication*, and *experimentation*. In *validation*, we compare traditional metrics of ED throughput to simulated output statistics to ensure the model is sufficiently accurate. The simulation also produces numerous statistics which are difficult to track in a real ED setting, but prove useful in understanding ED process of care. In *explication*, we use minute-by-minute tracking of resource utilization, patient locations, and acuity-specific door-to-event statistics to identify causes of delays in ED throughput. Finally, we conducted a series of rigorous *numerical experiments* to test the effectiveness of introducing additional resources, implementing alternate care pathways, and reducing boarding delays in both the nationally average and average academic ED settings.

### Validation: the model provides consistent and faithful outputs

We constructed and tuned the model to match nationally average data. After appropriately updating data-driven parameters such as patient arrivals, patient complexities and ED resource levels, we modified the mean lab turnaround time from 30 minutes to 45 minutes and the mean imaging turnaround time from 75 minutes to 90 minutes based on ED provider estimates. The result closely approximated average throughput metrics from the academic hospital survey (Table 1).

We report our results for each environment in terms of the outcome of 30-day simulations. The output statistics we used for validation are LWBS rate, door-to-doctor, door-to-disposition, door-to-exit, patients per doctor per hour, and patients per bed per year. The standard deviations reported in the Outputs section of Table 1 reflect variation in the monthly average statistics. We stress that whereas the statistics from the upper half of Table 1 are directly input to the model as parameters, the reported Output benchmarks are outcomes of the simulation.

To check for consistency with other existing models, we compared our results to those reported by Khare et al. [18], who published a model using parameters characterizing acuity-dependent arrivals, LWBS tolerance, treatment lengths, and admit rates. Accounting for ED beds, physicians, and boarding times, they concluded that reducing boarding times by 25% decreased mean length of stay by 22 minutes, while five additional beds increased mean length of stay by seven minutes. Using their input parameters, we were able to simulate an ED with a similar mean length of stay and LWBS rate, and then replicate their results: our model predicted that reducing boarding times by 25% reduced mean length of stay by 23.4 minutes, while five additional beds (and sufficient nursing coverage) had no effect on mean length of stay.

### Explication: identifying site-specific causes of crowding

**Figure 3:**
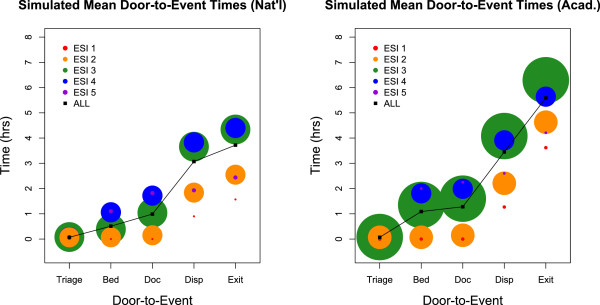
**Simulated door-to-event times.** The radius of each dot corresponds to the number of patients in that demographic and the sizes are comparable across plots. The timeliness of care delivery is largely affected by patient acuity.

Our model produces a novel breakdown of well-known statistical benchmarks in terms of patient acuity. In Figure 3, we display key door-to-event times in both the nationally average and the average academic environments. The colors indicate patient acuity and the radii of the circles is proportional to the absolute number of simulated patients treated in that acuity group. Figure 3 reaffirms that the timeliness of care delivery is highly acuity-dependent [35]. Whereas ESI-1 and ESI-2 patients are treated efficiently, lower acuity patients often experience tremendous delays. For example, ESI-3 patients in the academic ED have an *average* length of stay over 6 hours (Figure 3, right panel, rightmost green circle). This is due to two properties of typical ESI-3 patients: low prioritization, which accounts for lengthy door-to-bed times, and widely-ranging complexities, which contribute to long doctor-to-exit times.In order to understand the causes of ED crowding, we examine Figure 2. In the bottom two panels, we see the simulated mean idle resources as a function of time of day. While there are essentially zero idle physicians from noon to midnight in the nationally average ED, beds are the limiting resource in the academic setting. The impact can be seen in the middle two panels. Note the sharp increase of patients in the waiting room in the academic setting every evening. This stems from a combination of a high influx of patient arrivals – particularly high-complexity ESI-2 and ESI-3 patients (see top two panels of Figure 2) – combined with a rise in the number of boarded patients. This dynamic makes plain why adding beds (or reducing boarding delays) can have a significant impact in an average academic environment, but little to no effect in a nationally average environment.

### Numerical experiments

#### Improvement from resource addition is site-specific

**Figure 4:**
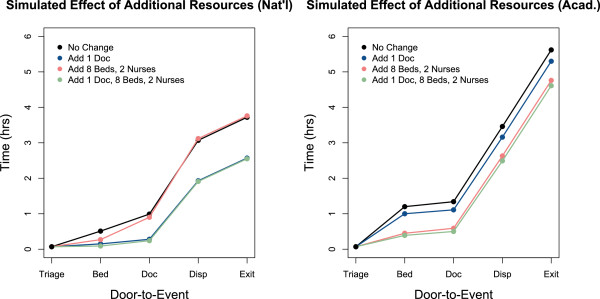
**Effect of additional resources on patient flow.** Simulated mean door-to-event times with additional resources for each ED environment. In both settings, a suite of resources is no more effective than a single, well-targeted resource.

Identifying the primary causes of crowding in an ED is a critical step in knowing how to increase throughput. Importantly, our model shows that extensive – but poorly-targeted – resource additions can have a negligible impact on patient flow. In Figure 4, we display simulated mean door-to-event times that result from a few resourcing remedies.

The response is different in the two settings. The addition of one full-time physician significantly reduced mean length of stay in the provider-limited national setting, but had little effect in the bed-limited academic setting. Conversely, additional beds and nurses significantly affected the mean length of stay in the academic setting, but not the national setting.We also observe that when an ED is primarily bottlenecked by a single resource, adding a combination of resources provides no more improvement than a highly targeted remedy. This is manifested in the left panel of Figure 4, where the door-to-event times that result from adding one doctor, eight beds, and two nurses is nearly identical to the result from adding one doctor alone. Furthermore, we note that the model can identify the point of diminishing returns. For example, we found that adding one doctor in the national setting reduces mean length of stay by one hour, but adding a second doctor does not further reduce mean length of stay (not depicted).

#### Fast track mechanisms can help *all* patients in provider-limited settings

Due to prioritization, low-acuity (ESI-4 and ESI-5) patients can often wait hours for what will be quick treatment and discharge from the ED. To expedite care for these patients, many EDs have implemented a Fast Track (FT) mechanism – a separate bay staffed with midlevel providers equipped to treat low-acuity patients. Often times to save on capital costs, beds and staff are repurposed from the MTA, leaving fewer resources available for MTA patients. Our model can quantify this tradeoff.

**Figure 5:**
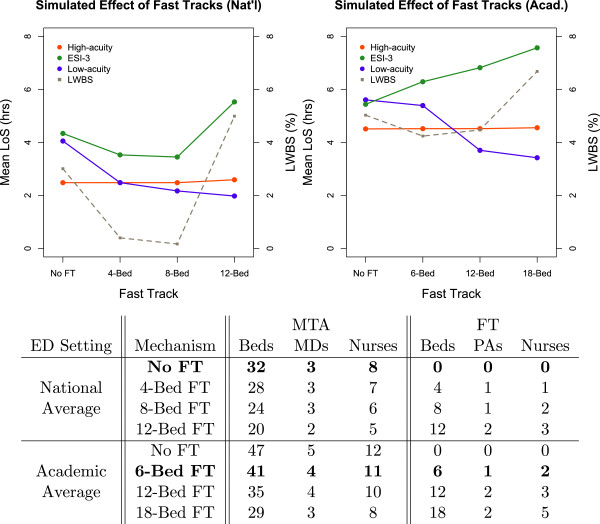
**Effect of fast track mechanisms on patient flow.** Simulated effect of Fast Track mechanisms in the nationally average and average academic environments. **Bold** values are the standard settings for each environment. FT mechanisms in the bed-limited academic setting are a clear tradeoff between low-acuity and ESI-3 throughput.

We measured the effect of various FT mechanisms on length of stay and LWBS. The number of beds repurposed for each FT were chosen so that the relative sizes were roughly equivalent between the national and academic settings. For example, a 4-Bed FT in the 32-bed national setting utilizes 12.5% of total bed capacity, and a 6-Bed FT in the 47-bed academic setting utilizes 12.8% of total bed capacity. In accordance with the ESI Implementation Handbook [27], ESI-4 and ESI-5 patients were eligible to use the fast track, and these patients were assigned to either the MTA or FT based on bed availability. Patients could not switch treatment areas once assigned, and providers only treated patients in the area to which the providers were assigned (other configurations are possible).The model demonstrates that there are settings where resources can be diverted to the FT without compromising care for higher acuity patients in the MTA. For example, in the provider-limited national setting, the 8-Bed FT most efficiently achieves this goal (left panel of Figure 5). Because low-acuity patients were rapidly treated in the FT, they tended not to occupy MTA beds, where their treatment was significantly delayed due to prioritization. This freed more beds for ESI-3 patients, reducing their mean length of stay. High-acuity (ESI-1 and ESI-2) patients were not affected.Observe that diverting too many resources to the FT adversely affects ESI-3 patients’ mean length of stay. In fact, there exist settings where any diversion of resources to the FT compromises care for patients in the MTA. In our model, ESI-3 patients in the academic setting experience an increased mean length of stay for all FT mechanisms (right panel of Figure 5).

#### Reducing boarding times can help *all* patients in bed-limited settings

Patients dispositioned to admit are boarded in the ED if a hospital bed is not available. These patients decrease ED capacity by effectively blocking a bed and occupying staff. We examined the effect of varying boarding times on throughput in both ED settings.

**Figure 6:**
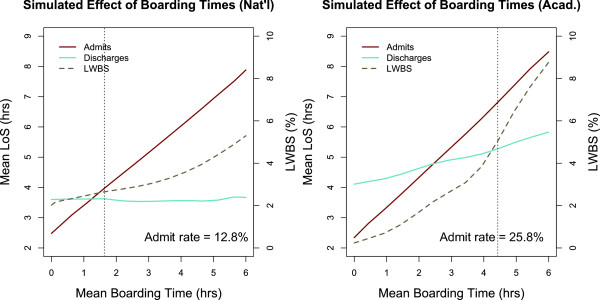
**Effect of reducing boarding times on patient flow.** Simulated effect of boarding times on length of stay and LWBS in the nationally average and average academic ED settings. The dotted vertical line marks the standard mean boarding time for each setting. The bed-limited academic setting has a higher admit rate and shows greater sensitivity to boarding times.

Figure 6 shows the sensitivity of the mean length of stay for admits, discharges, and overall LWBS rate to mean boarding time in each setting. As expected, we see that the mean boarding time directly affects the mean length of stay for admits in both settings. However, in the national setting, the mean length of stay for discharges and the overall LWBS rate is not largely affected by changing boarding times. This is because discharges and LWBS patients (who tend to be low-acuity) are affected by the number of blocked beds, which is a function of both boarding times *and admit rate*. The national setting is not primarily bed-limited and has a relatively low admit rate of 12.8%. Thus, systematically reducing boarding times unblocks a small number of beds in a setting where beds are not scarce to begin with.

In the academic setting, on the other hand, we observe a well-known phenomenon [22,23,26,36] that longer boarding times increase ED crowding. The right panel of Figure 6 quantifies how much the mean length of stay for discharges and overall LWBS rate decrease as boarding times are reduced. This is because the academic setting has a relatively high admit rate of 25.8% and is bed-limited. Thus, lowering boarding times unblocks a significant number of beds in an environment where crowding is primarily caused by bed availability. This suggests that in this setting, systematically reducing boarding times can be effective in improving ED throughput.

The dynamics of hospital capacity throughout the day largely determine ED boarding times. This phenomenon may be affected by the timing of hospital discharges and can be quantified by this model. Due to a lack of available data, we do not present this here.

## Discussion

Emergency Department crowding is a complex problem affecting more than 130 million patient visits per year in the U.S. [36]. Although emergency departments play a vital role in providing unscheduled access to healthcare, financial strain and limited resources challenge hospitals to provide timely and effective care. Computer simulation of ED patient flow can capture the inherent complexity of this system and elucidate its underlying dynamics. With this goal in mind, we have developed a flexible model of ED patient flow. In the span of minutes (on a personal laptop), we can accurately simulate ED throughput across a wide variety of settings. The model generates a minute-by-minute census of patients in the ED (Figure 2, bottom panels), and calculates traditional throughput metrics broken down by both acuity (Figure 3) and disposition (Figure 6).

As expected, when the model is equipped with realistic parameters, we see pervasive ED crowding. In the current world of scarce resources and little margin for error, it is essential to rigorously identify the specific causes of crowding, so that targeted management interventions can have maximal effect. Our model can predict and quantify how a particular ED will respond to a given “what if” scenario.With our ability to generate non-traditional patient flow statistics, such as a minute-by-minute account of idle resources (Figure 2, middle panels), we can weight the various factors that cause crowding on a site-to-site basis. One of the recurring observations in our investigation is that each simulated environment has its own dominant resource bottleneck. Further highlighting the importance of quantified predictions, we found that adding a suite of resources is no more effective than adding a single, well-targeted resource (Figure 4).

To demonstrate this phenomenon, we constructed and then investigated two qualitatively distinct ED environments: one created from statistics averaged over all U.S. emergency departments, and another from statistics averaged over a cohort of U.S. *academic* emergency departments (Figure 1). We found that a shortage of providers dictated crowding in the nationally average setting, but a shortage of beds was the primary cause of delays in the average academic environment (Figure 2). As a result, solutions that aim to increase bed capacity – such as systematically reducing boarding times – are effective in the average academic environment, but limited in the nationally average setting (Figure 6). Fast Track mechanisms had the converse effect (Figure 5).

In this way, simulation of EDs does more than confirm management intuitions. Having a comprehensive view of patient flow can help construct a system-wide understanding of what given management interventions actually accomplish. For example, the observation that the response to management interventions is highly sensitive to local resource limitations is perhaps undervalued in some data-driven assessments of ED performance. A 2008 report from the American College of Emergency Physicians (ACEP) concluded that, “the clearest cause of crowding [in the ED] is the boarding of admitted patients”, and warned that separate fast track mechanisms, “will create silos and obstacles to patient flow” [37]. However, a 2011 survey report from the National Association of Public Hospitals (NAPH) lists the implementation or expansion of a fast track mechanism as a “high performance strategy” to improve ED throughput [38]. Moreover, the NAPH recommends expanding ED bed space – a strategy Khare et al., whose simulation parameters produced a provider-limited setting, found to be ineffective [18]. We note that the NAPH survey sampled from its membership, which features a large number of community and safety-net hospitals that resemble our nationally average ED. It is then consistent that they would find fast track mechanisms to be useful. On the other hand, it is not clear which hospitals contributed to the conclusions from ACEP. If the sample is skewed toward large hospitals with a more acute patient mix, it would help explain these contradicting recommendations.

## Conclusion

We have constructed, and made publicly available at http://spark.rstudio.com/klopiano/EDsimulation/, an efficient model of ED patient flow which captures the complexities of ED process of care. With the flexibility of numerous input parameters, our model can accurately simulate a wide variety of environments. We investigated two qualitatively distinct ED environments and found that similar changes to process of care – such as adding resources, implementing fast track mechanisms, or systematically reducing boarding times – had very different effects on patient flow. Accurately predicting the effects of these changes is often difficult, which suggests the usefulness of more granular simulations in understanding ED dynamics. Moreover, our model’s ability to accurately quantify these dynamics provides a means to identify specific bottlenecks and test the effects of proposed operational changes. This can allow ED and hospital managers to formulate cost-effective, hospital-specific solutions to ED crowding.

## Competing interests

The authors declare that they have no competing interests.

## Authors’ contributions

JK and JAT conceived the study. JEH, JL, and SAM designed and wrote the preliminary simulation code. JEH wrote the final simulation code. JEH and JL researched publicly available data, and estimated input parameters. JEH, JL, and SAM designed and analyzed preliminary numerical experiments. JEH performed the final numerical experiments. SAM, JK, and JAT provided expert advice on study design and oversaw the project. JEH, SAM, and JAT drafted the manuscript, and all authors contributed to its revision. JEH and KKL adapted the simulation code to the online platform. JEH takes responsibility for the paper as a whole. All authors read and approved the final manuscript.

## Pre-publication history

The pre-publication history for this paper can be accessed here:

http://www.biomedcentral.com/1472-6947/14/50/prepub
